# Changes in Trophic Groups of Protists With Conversion of Rainforest Into Rubber and Oil Palm Plantations

**DOI:** 10.3389/fmicb.2019.00240

**Published:** 2019-02-12

**Authors:** Garvin Schulz, Dominik Schneider, Nicole Brinkmann, Nur Edy, Rolf Daniel, Andrea Polle, Stefan Scheu, Valentyna Krashevska

**Affiliations:** ^1^Johann-Friedrich-Blumenbach Institute for Zoology and Anthropology, University of Göttingen, Göttingen, Germany; ^2^Department of Genomic and Applied Microbiology and Göttingen Genomics Laboratory, University of Göttingen, Göttingen, Germany; ^3^Department of Forest Botany and Tree Physiology, University of Göttingen, Göttingen, Germany; ^4^Department of Agrotechnology, Faculty of Agriculture, Tadulako University, Palu, Indonesia; ^5^Centre of Biodiversity and Sustainable Land Use, University of Göttingen, Göttingen, Germany

**Keywords:** high-throughput sequencing, V4 region of 18S rRNA, soil protist diversity, community composition, rainforest transformation, Indonesia

## Abstract

Protists, abundant but enigmatic single-celled eukaryotes, are important soil microbiota providing numerous ecosystem functions. We employed high-throughput sequencing of environmental DNA, targeting the V4 region of the 18S rRNA gene, to characterize changes in their abundance, species richness, and community structure with conversion of lowland rainforest into rubber agroforest (jungle rubber), and rubber and oil palm plantations; typical agricultural systems in Sumatra, Indonesia. We identified 5,204 operational taxonomic units (OTUs) at 97% identity threshold of protists from 32 sites. Protists species richness was similar in rainforest, jungle rubber and oil palm plantations but significantly lower in rubber plantations. After standardization, 4,219 OTUs were assigned to five trophic groups, and inspected for effects of land-use change, and potential biotic and abiotic driving factors. The most abundant trophic group was phagotrophs (52%), followed by animal parasites (29%), photoautotrophs (12%), plant parasites (1%), and symbionts (<1%). However, the relative abundance and OTU richness of phagotrophs and photoautotrophs increased significantly with increasing land-use intensity. This was similar, but less pronounced, for the relative abundance of symbionts. Animal and plant parasites decreased significantly in abundance and species richness with increasing land-use intensity. Community compositions and factors affecting the structure of individual trophic groups differed between land-use systems. Parasites were presumably mainly driven by the abundance and species richness of their hosts, while phagotrophs by changes in soil pH and increase in Gram-positive bacteria, and photoautotrophs by light availability. Overall, the results show that relative species richness, relative abundance, and community composition of individual trophic groups of protists in tropical lowland rainforest significantly differ from that in converted ecosystems. This is likely associated with changes in ecosystem functioning. The study provides novel insight into protist communities and their changes with land-use intensity in tropical lowland ecosystems. We show, that trophic groups of protists are powerful indicators reflecting changes in the functioning of ecosystems with conversion of rainforest into monoculture plantations.

## Introduction

Tropical rainforests are one of the most threatened ecosystems in the world (Koh et al., 2011; Wilcove et al., 2013). Through high demand for cropland due to an increasing human population, large areas suffer from deforestation or peatland degradation and are converted into agricultural systems (Gibbs et al., 2010; Miettinen et al., 2013; Margono et al., 2014). This is especially true for Indonesia, one of the world’s top producers and exporters of palm oil and rubber (Koh et al., 2011; Marimin et al., 2014). On Sumatra alone, approximately 12 million ha of tropical rainforest have been converted into oil palm and rubber plantations since the 1980s (Laumonier et al., 2010). Conversion of rainforest into agricultural land strongly reduced the abundance and species richness of animals and plants (Barnes et al., 2014; Drescher et al., 2016; Rembold et al., 2017). As a result, ecosystem functions of the converted tropical systems, such as carbon storage, air quality, flood and drought prevention, decomposition, and nutrient cycling, are changing (Sala et al., 2000; Dislich et al., 2017; Guillaume et al., 2018; Krashevska et al., 2018).

Ecosystem functions depend to a large extent on the functional diversity of the belowground system (De Deyn et al., 2003; Bardgett and van der Putten, 2014; Fierer, 2017). Virtually all biogeochemical cycles are driven by soil microbial communities, and microorganisms play a key role in decomposing soil organic matter and mineralizing the nutrients therein (Falkowski et al., 2008; Delmont et al., 2012). Despite the importance of microorganisms in the belowground system, their diversity and functions are still poorly studied. Microorganisms in soils are represented by archaea, bacteria, fungi and protists, but the latter often are overlooked in soil ecology studies. Protists, eukaryotic single-celled organisms, are neither animals, nor plants, nor fungi, but make up the majority of all eukaryotic life forms (Adl et al., 2012; Geisen et al., 2018). They are not only highly diverse in species richness but also in life cycles, trophic interactions, and cellular structures. They can obtain carbon photoautotrophically and heterotrophically, form symbiotic relationships with animals, plants and fungi, parasitize and be parasitized by other protists (Kinne and Lauckner, 1980; Saleem et al., 2016; Geisen et al., 2018). They can reach densities of up to 100,000 individuals per gram of soil (Geisen and Bonkowski, 2017). However, their diversity in soil is very different to that of aquatic ecosystems, where they are best studied, but still is underestimated; protist diversity has been seen as “near imponderable” (Foissner, 1999b). This has been confirmed recently by high throughput sequencing (HTS) methods (Bates et al., 2013; Richardson et al., 2014; Geisen et al., 2015c; Geisen, 2016; Grossmann et al., 2016). High throughput sequencing approaches are promising in multiple ways. First, they improve access to the cryptic belowground biodiversity. They avoid the problem in single-celled organisms that the majority of taxa are difficult to extract and cultivate, and that enrichment cultures introduce a bias in abundance and species richness estimates (Geisen et al., 2015c). High throughput sequencing of soil environmental DNA (eDNA) has successfully been used to examine the biodiversity and structure of protist communities along environmental gradients in Canada (Heger et al., 2018), Switzerland (Seppey et al., 2017), Costa Rica, Panama, and Ecuador (Mahé et al., 2017), and the results suggest that protists, but not arthropods, are the most diverse eukaryotes in tropical rainforests. However, in spite of the fast increase in HTS data and information in reference databases, knowledge on protists in Southeast Asia is scarce and studies on protists of tropical land-use systems are lacking entirely.

To assess the effect of rainforest conversion on protists and to identify driving factors for their community composition, we used Illumina MiSeq HTS of soil eDNA to measure abundance and species richness of protists in tropical rainforest, rubber agroforests (jungle rubber), rubber plantations and oil palm plantations in Sumatra, Indonesia. To understand community functions of the vast diversity of protists recovered by this method, OTUs were categorized into five trophic groups; phagotrophs, photoautotrophs, animal parasites, plant parasites, and symbionts. Using these broad categories, we compared community composition between land-use systems, i.e., from low to higher management intensity. The rainforest was used as reference system of low anthropogenic influence. Jungle rubber is a traditional managed agroforest system, where rubber trees (*Hevea brasiliensis*) are interspersed with native tree species. Both plantation systems, rubber and oil palm (*Elaeis guineensis*), represent intensively managed monocultures with high fertilizer input including liming, for details see Kotowska et al. (2015) and Drescher et al. (2016). Finally, we inspected potential explanatory factors driving the distribution of the different trophic groups between land-use systems, including biodiversity of plants and animals, and soil phospholipid fatty acids (PLFAs) as proxies for bacterial and fungal communities, and abiotic environmental factors (Krashevska et al., 2015; Drescher et al., 2016).

Based on previous studies on macro-, meso- and microfauna, as summarized in Clough et al. (2016) and Drescher et al. (2016), we hypothesized that (1) protists are less diverse in more intensively managed land-use systems, i.e., decline in species richness from rainforest to oil palm plantations, and that (2) trophic groups of protists are differentially affected by land-use intensification. In more detail, we expected that in oil palm plantations, with higher bacterial abundance (Schneider et al., 2015), phagotrophs increase in species richness and abundance, and with increasing canopy openness in plantations (Drescher et al., 2016), photoautotrophs increase. Further, we expected the abundance and species richness of parasites and symbiotic protists to follow their host availability and therefore decrease in plantations.

## Materials and Methods

### Study Sites and Sampling

The sampling sites were located in the tropical lowlands of the Jambi Province in Sumatra, Indonesia. Two landscapes were studied, Bukit Duabelas (2° 0′ 57” S, 102° 45′ 12” E) and Harapan (1° 55′ 40” S, 103° 15′ 33” E). At each landscape four typical land-use systems representing the conversion from rainforest into plantations with increasing land-use intensity were selected: secondary lowland rainforest (rainforest), rubber agroforest (jungle rubber), rubber plantation (rubber) and oil palm plantation (oil palm). Each land-use system was replicated four times in each landscape, resulting in 32 sampling sites with three sub-plots each.

Samples were taken in October/November 2012 (jungle rubber, rubber plantations and oil palm plantations) and November/December 2013 (rainforest) as described in Sahner et al. (2015). In short, in each subplot five soil cores (4 cm diameter and 20 cm depth) were taken. Coarse roots and stones (>5 mm) were removed by consecutive sieving through 10 and 5 mm mesh. The samples from each subplot were pooled and homogenized resulting in one bulk soil sample per subplot resulting in 96 samples in total. Reaction tubes (50 ml, Sarstedt, Nümbrecht, Germany) with bulk soil were opened, a gauze was added to avoid soil loss during freeze drying and the samples were precooled for at least 3 h in a −80°C freezer. Freeze drying was conducted in a VirTis Benchtop K Freeze Dryer (SP Industries, Warminster, PA, United States) with a dual stage rotary vane vacuum pump (Trivac E2, Leybold Vakuum GmbH, Köln, Germany) for approximately 32 h. After freeze drying, three perforated Eppendorf tubes filled with 5 g of silica gel (Carl Roth, Karlsruhe, Germany) were added to the reaction tubes to keep the soil samples dry before shipping to the University of Göttingen.

### Permission

The Ministry of Research and Technology RISTEK (Kementrian Ristek dan Teknologi, Jakarta, Indonesia) provided the research permit (Kartu Izin Peneliti Asing, permission number: 333/SIP/FRP/SM/IX/2012). The Research Center for Biology of the Indonesian Institute of Science LIPI (Lembaga Ilmu Pengetahuan Indonesia, Jakarta, Indonesia) recommended issuing a sample collection permit [Rekomendasi Ijin Pengambilan dan Angkut (SAT-DN) Sampel Tanah dan Akar, number: 2696/IPH.1/KS:02/XI/2012]. The collection permit (number: S.16/KKH-2/2013) and an export permit (reference number: 48/KKH-5/TRP/2014) were provided by the Directorate General of Forest Protection and Nature Conservation PHKA (Perlindungan Hutan dan Konservasi Alam, Jakarta, Indonesia) under the Ministry of Forestry of the Republic of Indonesia. The Chamber of Agriculture of Lower Saxony (Plant Protection Office, Hanover, Germany) provided the import permits (Letter of Authority, numbers: DE-NI-12-69 -2008-61-EC, DE-NI-14-08-2008-61-EC).

### DNA Extraction and Amplification

DNA was extracted using the MoBio PowerSoil isolation kit (Dianova, Hamburg, Germany) as recommended by the manufacturer. The hypervariable V4 region of the 18S rRNA gene was amplified using the general eukaryotic primers TA-Reuk454FWD1 (5′-CCAGCASCYGCGGTAATTCC-3′) and TA-ReukREV3 (5′-ACTTTCGTTCTTGATYRA-3′) (Stoeck et al., 2010) paired with the MiSeq-Adapters Forward overhang (5′-TCGTCGGCAGCGTCAGATGTGTATAAGAGACAG) and Reverse overhang (5′-GTCTCGTGGGCTCGGAGATGTGTATAAGAGACAG). For amplification, the Phusion High Fidelity DNA Polymerase kit (Thermo Fisher Scientific, Germany) was used. The PCR reaction mixture contained 10 μl of fivefold Phusion GC Buffer, 1 μl of the forward and reverse primers (10 μM), 1 μl MgCl_2_ (50 mM), 1 μl dNTPs (10 mM), 2.5 μl DMSO, 0.5 μl Phusion Polymerase (1 U) and 1 μl template DNA. The following thermocycling scheme was used for amplification: initial denaturation at 98°C for 1 min, 35 cycles of denaturation at 98°C for 30 s, annealing at 60°C for 45 s and extension at 72°C for 1 min, followed by a final extension period at 72°C for 5 min. Amplicon length was approximately 400 bp. All amplicon PCRs were performed three times and pooled equimolar for sequencing. The University of Göttingen Genomic Laboratory facility determined the sequences of the 18S amplicons using MiSeq.

### Sequence Data Deposition

The 18S rRNA gene sequences were deposited in the European Bioinformatics Institute (EMBL-EBI) European Nucleotide Archive (ENA) under the study accession number PRJEB23943.

### Bioinformatic Analysis of 18S rRNA Gene Sequences

The resulting 18S rRNA gene sequences were processed and analyzed employing PEAR, cutadapt, USEARCH 9.24 and QIIME 1.9.1 (Caporaso et al., 2010). Initially, sequences shorter than 250 bp, containing unresolved nucleotides, exhibiting an average quality score lower than 20, were removed with split_libraries_fastq.py. Additionally, we used cutadapt (Martin, 2011) with default settings for efficient forward and reverse primer removal. Chimeric sequences were removed using UCHIME2 (Edgar et al., 2011) with SSU SILVA 128 as a reference dataset.

Operational taxonomic unit (OTU) determination was performed at a genetic divergence of 3% (species level) with USEARCH. Taxonomic classification was performed with parallel_assign_taxonomy_blast.py against the same database. OTU tables were created using USEARCH. Singletons, bacteria, archaea, chloroplasts, metazoa, Streptophyta, fungi and unclassified OTUs were removed from the table by employing filter_otu_table.py (quality-filtered data). In order to homogenize the differences in the number of reads per sample, we randomly selected 2,300 sequences for each sample (standardized data with 4,219 OTUs). Diversity estimates and rarefaction curves were generated by employing alpha_rarefaction.py (Supplementary Figure 1).

### Data Analysis

Data handling and transformation was done with the packages *dplyr* (Wickham et al., 2017), *reshape2* (Wickham, 2007), and *tidyr* (Wickham and Henry, 2017) in R (R Core Team, 2017). Graphics were implemented in R with the additional packages *ggplot2* (Wickham, 2009), *ggrepel* (Slowikowski, 2017), *ggpubr* (Kassambara, 2017), *scales* (Wickham, 2017), *vegan* (Oksanen et al., 2017), and *viridis* (Garnier, 2018).

After data standardization procedure, 4,219 OTUs were categorized into five trophic groups (symbionts, photoautotrophs, phagotrophs, plant parasites and animal parasites) based on the work of Adl and Gupta (2006), Seppey et al. (2017), and Geisen et al. (2018) (Supplementary Table 1). All OTUs that could not be ascribed to one of these groups were categorized as undetermined. As the HTS approach is based on extracted DNA of soils, our data might include OTUs derived from extracellular DNA or encysted cells.

MANOVA as implemented in the *stats* package in R (R Core Team, 2017), including all five trophic groups showed that the relative abundance (Wilks’ λ = 0.2, *F*_3,91_ = 10.5, *p* < 0.001) and relative species richness (Wilks’ λ = 0.1, *F*_3,91_ = 29.6, *p* < 0.001) of trophic groups varied with land-use changes. Therefore, the effect of forest conversion on each of the trophic groups was analyzed separately using linear mixed-effects models with landscape (Harapan, Bukit Duabelas) as block, land-use (rainforest, jungle rubber, rubber, oil palm) as fixed effect and replicate plots and subplots fitted as random effect (Crawley, 2007), as implemented in the *nlme* package in R (Pinheiro et al., 2017). Tukey’s HSD test, as implemented in the *multcomp* package in R (Hothorn et al., 2008), was used to identify significant differences between means.

Discriminant function analysis (DFA), as implemented in STATISTICA 13.1 for Windows (StatSoft, Tulsa, OK, United States), was used to identify effects of the land-use system on overall protist communities (based on abundances of quality filtered data) and for each individual trophic group (based on relative abundance). Squared Mahalanobis distances (MD^2^) between group centroids were determined to identify significant differences in protist community structure between land-use systems.

Relationships between OTUs (based on relative abundance) and environmental factors were analyzed using distance-based redundancy analysis (db-RDA) with Bray-Curtis dissimilarity as distance measure as implemented in CANOCO 5.02 (Ter Braak and Šmilauer, 2012). RDA was chosen as the length of gradient of OTUs data was 3.10 SD units (Leps and Smilauer, 2003). The forward selection procedure of db-RDA allowed to relate OTUs (dependent variables) to a set of environmental factors (independent variables) by direct ordination. Environmental factors included water content, microbial basal respiration, microbial biomass, pH, C concentration, N concentration, air temperature, humidity, canopy openness, plant abundance, plant richness, soil fauna abundance, soil fauna richness, the sum of PLFAs relative markers for Gram-positive bacteria (i15:0, a15:0, i16:0, i17:0), Gram-negative bacteria (16:1ω7, cy17:0, cy19:0), fungi (18:2ω6,9, 18:3ω6, 18:3ω3) and neutral lipid acid marker for arbuscular mycorrhizal fungi (16:1ω5c). The data for the analyses were taken from Krashevska et al. (2015), Drescher et al. (2016), and A. Potapov (unpublished data), for details see Supplementary Table 2. Monte Carlo tests (999 permutations) were performed to evaluate the significance of individual axes (Ter Braak, 1996).

## Results

Sequencing and quality filtering resulted in 2,433,278 high-quality 18S rRNA gene sequences from all subplots. After removal of singletons, bacteria, archaea, chloroplasts, metazoa, Streptophyta, fungi and unclassified OTUs, the dataset comprised 5,204 OTUs at 97% genetic identity and 220,800 sequences (quality-filtered data; for details see Supplementary Table 3). After subsampling (2,300 sequences per sample), the dataset comprised 4,219 OTUs, with an average number of 269 ± 65 OTUs per plot ranging from 132 (HR1) to 396 OTUs (BO1).

### Overall Protist Species Richness and Abundance

Protist OTU richness (based on quality-filtered data) differed significantly between land-use systems (*F*_3,27_ = 5.4, *p* = 0.005). It was similar in oil palm plantation (492 ± 145), jungle rubber (482 ± 156) and rainforest (459 ± 195), but significantly lower in rubber plantation (285 ± 94). Also, mean OTU richness was significantly higher in Bukit Duabelas (481 ± 154) as compared to Harapan landscape (378 ± 174; block effect *F*_3,27_ = 6.1, *p* = 0.02). In contrast to richness, total OTU abundance did not differ significantly between land-use systems with an overall mean of 25,347 ± 1,900 sequences (*F*_3,27_ = 0.3, *p* > 0.05). Neither Shannon nor Simpson diversity index differed between land-use systems (overall mean of 4.42 ± 0.44, *F*_3,27_ = 1.5, *p* = 0.23, and 0.96 ± 0.04, *F*_3,27_ = 0.9, *p* = 0.46, respectively).

**FIGURE 1:**
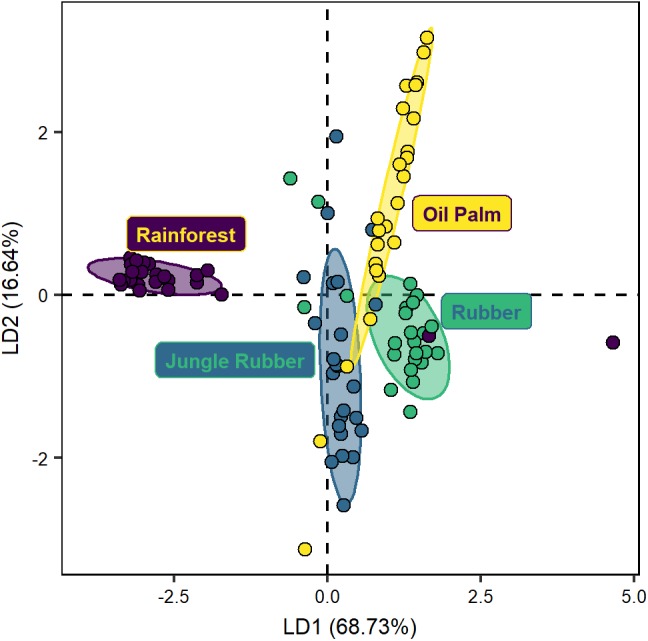
Discriminant function analysis of protist communities of four land-use systems (rainforest, jungle rubber, rubber plantation and oil palm plantation; Wilks’ λ = 0.12, *F*_12,235_ = 23.5, *p* < 0.001) based on quality-filtered data. Eigenvalues: LD1 = 0.60, LD2 = 0.25. Ellipses drawn for better visualization of the respective land-use systems include 75% of the respective plots.

The DFA separated protist community composition along land-use systems (Wilks’ λ = 0.1, *F*_12,235_ = 23.5, *p* < 0.001; Figure 1). The three linear discriminant functions explained 68.7%, 16.6%, and 14.6% of the variation, respectively. Protist communities in rainforest were separated from those in oil palm (MD^2^ = 38.6, *p* < 0.001) and rubber plantations (MD^2^ = 27.7, *p* < 0.001), and less pronounced also from those in jungle rubber (MD^2^ = 4.5, *p* < 0.001). Protist community composition in jungle rubber was most similar to that in rubber plantations (MD^2^ = 10.6, *p* < 0.001) and more separate from that in oil palm plantations (MD^2^ = 17.5, *p* < 0.001). The communities in rubber and oil palm plantations were also distinct (MD^2^ = 1.1, *p* < 0.001).

### Relative Abundance, Species Richness, and Community Composition of Trophic Groups

**FIGURE 2:**
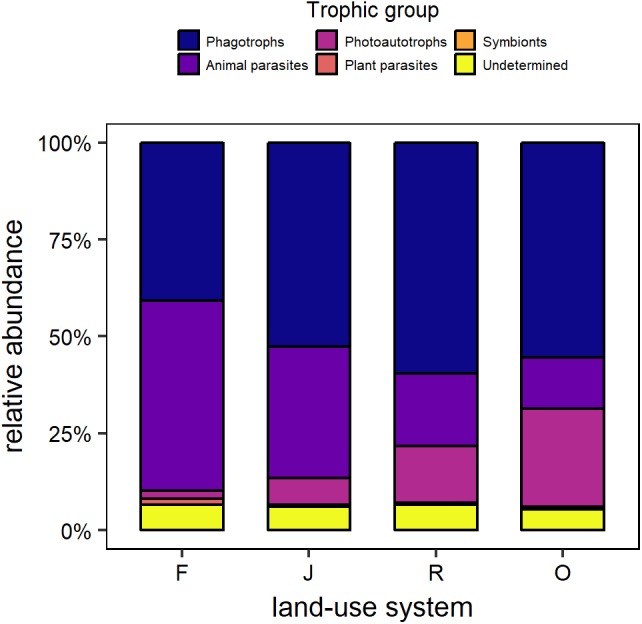
Relative OTU abundance of trophic groups of protists in the soil of rainforest (F), jungle rubber (J), rubber plantations (R), and oil palm plantations (O), based on standardized data.

Overall, the relative abundance of trophic groups declined in the order phagotrophs (52.1%), animal parasites (28.7%), photoautotrophs (12.2%), plant parasites (0.8%), and symbionts 0.1%); based on standardized data. About 6% of all sequences could not be assigned to any trophic group and were grouped as “undetermined.” Generally, in rainforest animal parasites and phagotrophs were the dominating groups, followed by undetermined, photoautotrophs, plant parasites and symbionts (Figure 2). In jungle rubber, phagotrophs were dominating, followed by animal parasites, photoautotrophs, undetermined, plant parasites and symbionts. Also, in rubber plantation phagotrophs dominated, followed by animal parasites, photoautotrophs, undetermined, plant parasites and symbionts. In oil palm plantation phagotrophs were dominant, followed by photoautotrophs, animal parasites, undetermined, plant parasites and symbionts.

#### Phagotrophs

**FIGURE 3:**
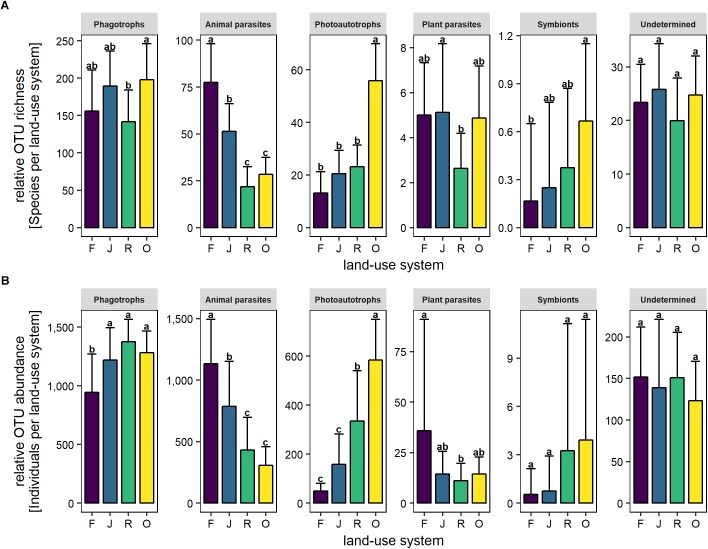
Relative OTU richness **(A)** and relative OTU abundance **(B)** of trophic groups of protists in soil of four land-use systems: rainforest (F), jungle rubber (J), rubber plantations (R), and oil palm plantations (O). Bars sharing the same letter do not differ significantly (Tukey’s HSD test, *p* < 0.05).

The relative OTU richness of phagotrophs was high in oil palm plantations, low in rubber plantations and intermediate in rainforest and jungle rubber (*F*_3,27_ = 4.1, *p* = 0.02; Figure 3A). By contrast, the relative OTU abundance of phagotrophs was similar in jungle rubber, and rubber and oil palm plantations, but significantly lower in rainforest (*F*_3,27_ = 9.2, *p* < 0.001; Figure 3B).

**FIGURE 4:**
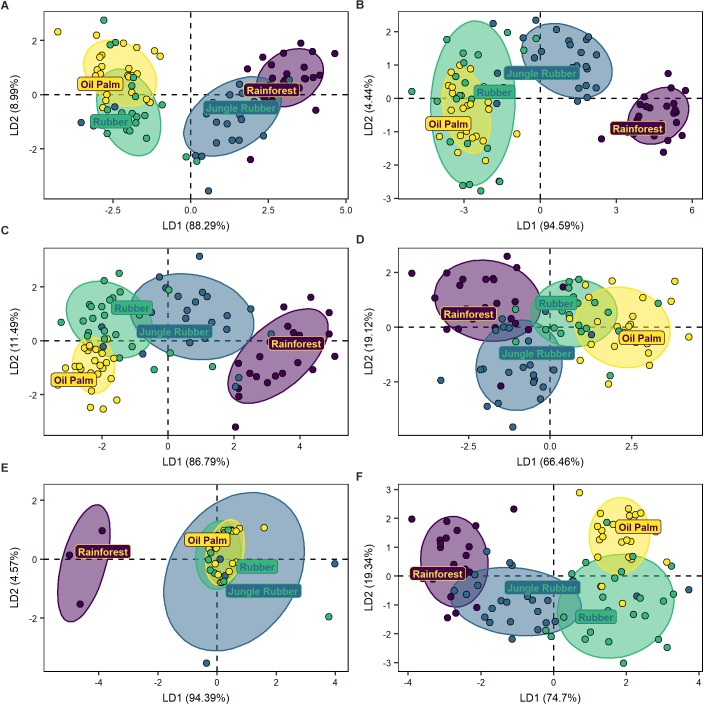
Discriminant function analyses of five trophic groups of protists **(A)** phagotrophs, **(B)** animal parasites, **(C)** photoautotrophs, **(D)** plant parasites, **(E)** symbionts, and of undetermined protists **(F)** from four land-use systems (rainforest, jungle rubber, rubber plantation, and oil palm plantation). Ellipses drawn for visualization of the respective land-use systems include 75% of the respective plots.

The DFA separated the communities of phagotrophs of the four land-use systems (Wilks’ λ = 0.2, *F*_12,235_ = 17.5, *p* < 0.001, Figure 4A). The community of phagotrophs in rainforest was separated from that in oil palm (MD^2^ = 21.0, *p* < 0.001) and rubber plantations (MD^2^ = 15.6, *p* < 0.001), and less pronounced also from that in jungle rubber (MD^2^ = 3.2, *p* < 0.001). Similar to total protists, the community composition of phagotrophs in jungle rubber was similar to that in rubber plantations (MD^2^ = 4.7, *p* < 0.001) and more distinct from that in oil palm plantations (MD^2^ = 9.0, *p* < 0.001). The communities in rubber and oil palm plantations also differed significantly (MD^2^ = 1.9, *p* < 0.001), but in part communities overlapped widely.

The dominance of phagotrophic species changed between land-use systems. Across all land-use systems the most dominant OTU was assigned to *Ischnamoeba* sp. (Variosea, Amoebozoa). It represented 8.1% of the protists relative abundance and reached 11.1% in rainforest, 10.0% in jungle rubber, 5.8% in rubber, and 6.4% in oil palm plantations. In rainforest *Bodomorpha* sp. (Sarcomonadea, Cercozoa) was the second dominant OTU followed by *Palpitomonas* sp. (Palpitea, Cryptista), BOLA868 (Tubulinea, Amoebozoa), *Lacrymaria* sp. (Litostomatea, Ciliophora), *Telonema* (Harosa, *incertae sedis*), and different *Heteromita* spp. (Sarcomonadea, Cercozoa). In jungle rubber, the dominance of OTUs differed from that in rainforest. Following *Ischnamoeba* sp., Discicristoidea (Nucletmycea) species were as abundant as *Palpitomonas* sp., followed by *Bodomorpha* sp., BOLA868, *Schizoplasmodium* sp. (Protostelea, Amoebozoa), *Platyophrya* sp. (Colpodea, Ciliophora), *Vermamoeba* sp. (Tubulinea, Amoebozoa), *Acanthamoeba* sp. (Discosea, Amoebozoa) and *Heteromita* sp. Similarly, in rubber plantations *Ischnamoeba* sp. was followed by Discicristoidea species, BOLA868, *Heteromita* spp., *Palpitomonas* sp., *Telonema* sp., another Glissomonadida, and a different Discicristoidea species. In oil palm plantations *Ischnamoeba* sp. was followed by *Heteromita* spp., *Telonema* sp., a member of Discicristoidea (different species than in jungle rubber), *Palpitomonas* sp., *Cercomonas* sp. (Sarcomonadea, Cercozoa), *Eocercomonas* sp. (Sarcomonadea, Cercozoa), and *Ceratomyxella* sp. (Protostelea, Amoebozoa).

#### Animal Parasites

The relative OTU richness of animal parasites was high in rainforest, lower in jungle rubber and lowest in rubber and oil palm plantations (*F*_3,27_ = 43.8, *p* < 0.001; Figure 3A). The relative OTU abundance of animal parasites followed a similar pattern (*F*_3,27_ = 22.1, *p* < 0.001; Figure 3B).

DFA separated animal parasite communities of the four land-use systems (Wilks’ λ = 0.1, *F*_12,235_ = 22.4, *p* < 0.001, Figure 4B). Animal parasite communities in rainforest were most distinct from those in oil palm (MD^2^ = 30.8, *p* < 0.001) and rubber plantations (MD^2^ = 28.2, *p* < 0.001), but less distinct from those in jungle rubber (MD^2^ = 5.3, *p* < 0.001). The animal parasite community in jungle rubber was separated from that in both oil palm (MD^2^ = 11.8, *p* < 0.001) and rubber plantations (MD^2^ = 9.3, *p* < 0.001), while the animal parasite communities in rubber and oil palm plantations differed little (MD^2^ = 0.9, *p* = 0.04).

In each of the land-use systems, the dominant animal parasites were species of the gregarines group (Gregarinomorphea, Miozoa). In rainforest the dominant OTUs included different *Gregarina* species. Another gregarine of the order Eugregarinorida was detected in rainforest but could not be identified further. Similar to rainforest, in jungle rubber gregarines were dominant. *Prismatospora* sp. and other not further identified species from the order Eugregarinorida were also detected. In rubber plantations an unspecified OTU belonging to Eimeriidae (Coccidiomorphea, Miozoa) was most abundant. Also, in rubber plantations different *Gregarina* spp. as well as *Syncystis* sp., *Psychodiella* sp. and *Monocystis* sp. were abundant. Similar to rubber plantations, the same unspecified OTU of the Eimeriidae family was most abundant in oil palm plantations, followed by *Monocystis* sp., *Psychodiella* sp. and other species of Eugregarinorida order but not *Gregarina* sp.

#### Photoautotrophs

The relative OTU richness of photoautotrophs was low in rainforest, jungle rubber and rubber plantations and significantly higher in oil palm plantations (*F*_3,27_ = 60.3, *p* < 0.001; Figure 3A). The relative OTU abundance followed a similar pattern but increased more linearly from rainforest to oil palm plantations (*F*_3,27_ = 30.8, *p* < 0.01; Figure 3B).

The DFA separated photoautotroph communities of the four land-use systems (Wilks’ λ = 0.2, *F*_12,233_ = 15.9, *p* < 0.001; Figure 4C). The community of photoautotrophs of rainforest was most distinct from the communities in rubber (MD^2^ = 17.9, *p* < 0.001) and oil palm plantations (MD^2^ = 17.5, *p* < 0.001), but less distinct from the community in jungle rubber (MD^2^ = 9.8, *p* < 0.001). The community of photoautotrophs in jungle rubber was more distinct from that in oil palm plantations (MD^2^ = 2.0, *p* < 0.001) than from that in rubber plantations (MD^2^ = 1.2, *p* = 0.001), with the latter differing only along the second axis (MD^2^ = 1.5, *p* = 0.003).

The most dominant photoautotroph in rainforest was *Ceratium* sp. from the Ceratiaceae family (Dinophyceae, Miozoa), followed by unspecified species of the family Cryptophycea, *Chrysochromulina* sp. from the Prymnesiaceae family (Prymnesiophyceae, Haptophyta), *Rhizosolenia* sp. from the Rhizosoleniaceae family (Bacillariophyceae, Ochrophyta) and unspecified chlorophytes species. In jungle rubber the Chlorophyta classes Chlorophyceae and Trebouxiophyceae were most dominant, followed by *Prymnesium* sp. (Prymnesiophyceae, Haptophyta), unspecified Dinoflagellata of the Dinophyceae family, *Bangia* sp. (Bangiophyceae, Rhodophyta) and *Ochromonas* sp. (Chrysophyceae, Ochrophyta). Rubber and oil palm plantations also were dominated by chlorophytes from the classes Chlorophyceae and Trebouxiophyceae. In the latter no species were identified, while Chlorophyceae included *Hylodesmus* sp., *Chlorosarcinopsis* sp. and *Bracteacoccus* sp.

#### Plant Parasites

The relative OTU richness of plant parasites was similar in rainforest, jungle rubber and oil palm plantations but lower in rubber plantations (*F*_3,27_ = 3.1, *p* < 0.001; Figure 3A). By contrast, the relative OTU abundance of plant parasites was similar in jungle rubber, rubber and oil palm plantations, but higher in rainforest (*F*_3,27_ = 3.4, *p* = 0.03; Figure 3B).

The DFA separated the plant parasite communities of the four land-use systems (Wilks’ λ = 0.5, *F*_12,227_ = 6.4, *p* < 0.001; Figure 4D). The plant parasite community in rainforest was most distinct from that in oil palm (MD^2^ = 6.8, *p* < 0.001) and rubber plantations (MD^2^ = 3.3, *p* < 0.001), but less from that in jungle rubber (MD^2^ = 1.2, *p* = 0.01). The plant parasite community of jungle rubber was distinct from that in oil palm plantations (MD^2^ = 3.0, *p* < 0.001). Further, the plant parasite communities differed between rubber and oil palm plantations but differences were less pronounced (MD^2^ = 0.9, *p* = 0.04). The plant parasite communities from jungle rubber and rubber plantation were not significantly distinct (MD^2^ = 2.3, *p* = 0.06).

The rainforest was dominated by Oomycetes, Oomycota: *Eurychasma* sp., *Pseudoperonospora* sp., *Olpidiopsis* sp., and *Phytophthora* sp. In jungle rubber, the dominating species were *Eurychasma* sp., *Olpidiopsis* sp. and *Pseudoperonospora* sp. followed by *Achlya* sp. and *Pythium* sp. (all Oomycetes, Oomycota), as well as *Sorodiplophrys* sp. and *Thraustochytrium* sp. (both Labyrinthulea, Bigyra). In rubber plantations *Eurychasma* sp. was most abundant, followed by *Pythium* sp., *Aphanomyces* sp., *Pseudoperonospora* sp., *Phytopythium sp.* and *Achlya* sp. In oil palm plantations *Eurychasma* sp. and *Aphanomyces* sp. (both Oomycetes, Oomycota) dominated followed *Polymyxa* sp. (Phytomyxea, Cercozoa), as well as unspecified Oomycetes and *Sorodiplophrys* sp. (Labyrinthulea, Bigyra).

#### Symbionts

The relative OTU richness of symbionts increased continuously with increasing land-use intensity from rainforest to oil palm plantations (*F*_3,27_ = 4.0, *p* = 0.02; Figure 3A). By contrast, the relative OTU abundance did not vary significantly between land-use systems (*F*_3,27_ = 2.2 and *p* > 0.05; Figure 3B).

The DFA separated the symbiont communities of the four land-use systems (Wilks’ λ = 0.3, *F*_12,69_ = 3.2, *p* < 0.001; Figure 4E). The symbiont community in rainforest was distinct from that in rubber (MD^2^ = 13.9, *p* < 0.001) and oil palm plantations (MD^2^ = 13.4, *p* < 0.001), but less from that in jungle rubber (MD^2^ = 10.1, *p* = 0.009). Further, the symbiont community in jungle rubber was distinct from that in oil palm plantations (MD^2^ = 3.9, *p* = 0.02), but neither symbiont communities between jungle rubber and rubber plantations (MD^2^ = 2.4, *p* = 0.07) nor between rubber and oil palm plantations differed significantly (MD^2^ = 0.1, *p* = 0.98).

In each land-use system, the dominating symbiont OTUs were different. However, all of them belonged to unspecified Syndiniales (Syndinea, Miozoa) in rainforest and jungle rubber also to *Saccinobaculus* sp. (Anaeromonadea, Metamonada).

#### Undetermined

The relative OTU richness of undetermined protists (*F*_3,27_ = 1.6, *p* > 0.05; Figure 3A) as well as the relative OTU abundance of undetermined protists did not vary significantly between land-use systems (overall mean 141 ± 62.7, *F*_3,27_ = 0.5, *p* > 0.05; Figure 3B).

The DFA separated the undetermined OTUs communities of the four land-use systems (Wilks’ λ = 0.2, *F*_12,235_ = 19.6, *p* < 0.001; Figure 4F). The undetermined community in rainforest was distinct from that in rubber (MD^2^ = 15.8, *p* < 0.001) and oil palm plantations (MD^2^ = 15.6, *p* < 0.001), but less from that in jungle rubber (MD^2^ = 1.9, *p* < 0.001). Further, the community in jungle rubber was distinct from that in oil palm (MD^2^ = 8.3, *p* < 0.001) and rubber plantations (MD^2^ = 8.2, *p* < 0.001) with the latter two also differing significantly (MD^2^ = 3.6, *p* < 0.001).

In each land-use system the undetermined protists were dominated by different Cercozoa, Stramenopiles and Amoebozoa. Neither of these could further be specified.

### Environmental Factors

**FIGURE 5:**
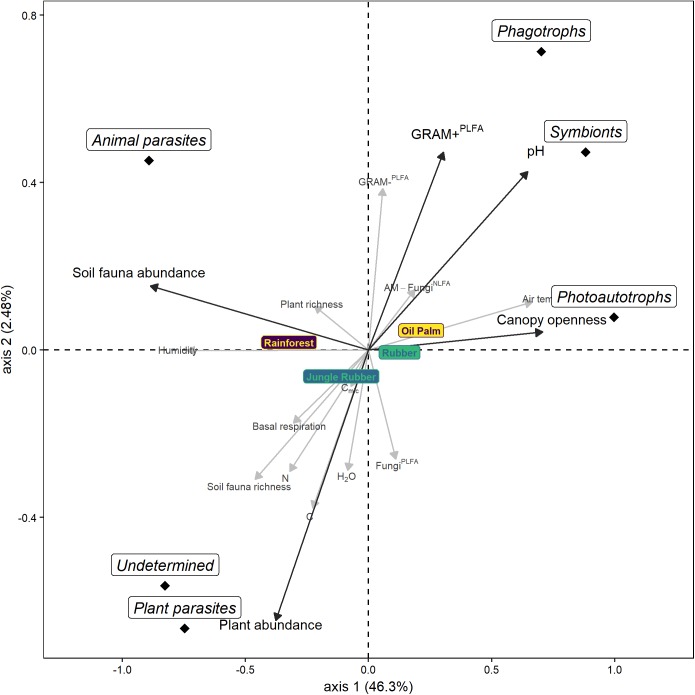
Distance-based RDA of protist OTUs in soil of four land-use systems (rainforest, jungle rubber, rubber, and oil palm plantation). Factors in black and bold are significant in the forward selection procedure and are used as constraining factors (soil pH, canopy openness, plant abundance, soil fauna abundance, the sum of Gram-positive bacterial phospholipid fatty acids = GRAM+^PLFA^), factors in gray are not significant (water content = H_2_O, microbial basal respiration = Basal respiration, microbial biomass = C_mic_, C concentration = C, N concentration = N, air temperature, humidity, plant richness, soil fauna richness, the sum of Gram-negative bacterial phospholipid fatty acids = GRAM-^PLFA^, the sum of fungal phospholipid fatty acids = Fungi^PLFA^ and relative marker of arbuscular mycorrhizal fungal neutral lipid acid = AM-Fungi^NLFA^). The position of trophic groups represents their centroid.

In the forward selection procedure of the db-RDA, five of the 17 environmental variables were significant (*p* < 0.05). These five variables explained 49.5% of the total variation: pH accounted for 33.5% (*F* = 47.3, *p* = 0.001), canopy openness for 9.2% (*F* = 14.9, *p* = 0.001), the sum of Gram-positive bacterial PLFAs for 3.2% (*F* = 5.5, *p* = 0.004), plant abundance for 1.8% (*F* = 3.2, *p* = 0.021) and soil fauna abundance for 1.8% (*F* = 3.1, *p* = 0.029). The first axis explained 46.3% of variation (*F* = 86.3, *p* = 0.001) and positively correlated with canopy openness, pH and the sum of PLFAs of Gram-positive bacteria but negatively with plant abundance and soil fauna abundance (Figure 5). The second axis only explained 2.5% of the variation (*F* = 6.3, *p* = 0.001) and positively correlated with the sum of Gram-positive bacterial PLFAs, pH, soil fauna abundance and canopy openness but negatively with plant abundance. The RDA separated the different land-use systems as well as the different trophic groups of protists. Phagotrophs and symbionts clustered with pH and the sum of Gram-positive bacterial PLFAs, photoautotrophs clustered with canopy openness, animal parasites with soil fauna abundance and plant parasites and undetermined protists with plant abundance.

## Discussion

Effects of land-use change on diversity and community composition of bacteria, archaea and various invertebrate groups have been investigated in soils of lowland Sumatra (Schneider et al., 2015; Clough et al., 2016; Drescher et al., 2016; Barnes et al., 2017; Klarner et al., 2017). However, information on protists, an abundant and diverse group of soil microbial eukaryotes, is lacking. Protists comprise a wide range of phylogenetic and trophic groups and are important determinants and indicators of ecosystem functioning. This study presents the first attempt toward understanding effects of land-use change on protist community structure and trophic assembly in tropical lowlands using high-throughput sequencing of eDNA.

### General Response

We hypothesized that protists are less diverse in more intensively managed land-use systems. Contrasting this hypothesis, the species richness of protists was only significantly reduced in rubber plantations, but similar in rainforest, jungle rubber and oil palm plantations. Seppey et al. (2017) also found the species richness of protists to differ little between forest, meadow and arable systems. In our study the Simpson index was high in each of the studied systems (mean 0.96 ± 0.04) indicating that protist diversity is high in each of the land-use systems investigated and little affected by conversion of rainforest into plantations. Contrasting these overall community characteristics, the community compositions of protists differed strongly between the four land-use systems, as indicated by the DFA. The overall high species richness but different community composition in the studied ecosystems reflects that protists comprise phylogenetically diverse groups of single cell eukaryotes with very different ecological traits and functions. Thus, to uncover changes in the functioning of protist communities associated with changes in land-use the community structure of individual trophic groups need to be considered.

### Trophic Groups

Contrasting protists in general, but conform to our hypothesis, the relative abundance and relative species richness of individual trophic groups of protists differed significantly between land-use systems. In addition, community composition of individual trophic groups of protists differed between land-use systems, suggesting that the community of each land-use system comprises specific protist taxa. For each trophic group of protists the community in rainforest was most distinct from that in the other land-use systems. The shift in community composition from rainforest to intensively managed land-use systems was represented by the first DFA axis, while the second axis separated protist communities of rubber and oil palm plantations. This pattern even applied to protists of undetermined trophic group. Separation of land-use systems, however, was less pronounced in symbionts, which likely is due to the low recovery of symbionts by the method used. Overall, the results support the notion of Grossmann et al. (2016) “that protistan community patterns are highly consistent with habitat types.”

**Phagotrophic protists** predominantly function as bacterial grazers (Clarholm, 1981, 2005), however, in part also as fungivores (Foissner, 1999a; Geisen et al., 2015a) or predators of other protists (Hess and Melkonian, 2014; Seppey et al., 2017) and even microfauna (Yeates and Foissner, 1995; Gilbert et al., 2000; Geisen et al., 2015b). Notably, species richness and abundance of phagotrophic protists increased in intensively managed land-use systems as compared to rainforest. By grazing on bacteria phagotrophic protists, in particular amoebae, increase the mobilization of bacterial nitrogen and thereby improve plant nutrition (‘microbial loop in soil’; Clarholm, 1994; Bonkowski, 2004; Rosenberg et al., 2009; Koller et al., 2013). However, due to fertilization plants are likely to invest less in supporting the microbial loop in plantations via root exudates, thereby detrimentally affecting the abundance of phagotrophic protists contrasting our observation. However, in addition to fertilizer input, plantations are limed and thereby soil pH is increased as compared to rainforest (Krashevska et al., 2015; Schneider et al., 2015). It often has been shown that pH is one of the main factors driving the structure of protist communities (Mitchell et al., 2013; Dupont et al., 2016; Lara et al., 2016), and this notion is supported by the increase in phagotrophs with increasing pH in plantations in the present study. Changes in protist communities with soil pH, however, are likely to be indirect via soil pH changing the structure of bacterial communities (Nicol et al., 2008; Schneider et al., 2015), which also influences the structure of protist communities (Saleem et al., 2013). In fact, phagotrophs also correlated with increased abundance of Gram-positive bacteria comprising potential prey, suggesting that phagotrophs function as bacterial grazers and this is particularly pronounced in plantations. This is consistent with findings of Schneider et al. (2015) documenting that bacteria and archaea thrive in rubber and oil palm plantations. Further, phagotrophs correlated closely with photoautotrophs, potentially reflecting that phagotrophs increasingly feed on algae in rubber and oil palm plantations, which is in line with recent findings (Seppey et al., 2017). Additionally, both positive correlations may also reflect that phagotrophs reduce the abundance of certain taxa of bacteria and these changes resulting in Gram-positive bacteria and photoautotrophs to thrive.

Representatives of phagotrophic protists, including bacterial grazers, algivores, and predators, formed part of the top ten most abundant OTUs in the studied rainforest and plantation systems. In each of the studies ecosystems the slow moving reticulose amoeboid grazer *Ischnamoeba* sp. was present, which is assumed to exclusively feed on bacteria (Berney et al., 2015). Further, *Telonema* spp. comprising algivore species was common in plantations. The two described members of this genus (*T. antarcticum* and *T. subtilis*) are common in marine and brackish waters (Vørs, 1992; Klaveness et al., 2005). They are apparently a diverse, deep branching member of chromistan lineage (Shalchian-Tabrizi et al., 2006, 2007) and are recently placed as sister group to the supergroup SAR forming the mega-assemblage TSAR (Strassert et al., 2019). To the best of our knowledge, we report *Telonema* sp. for the first time in soils. In rainforest an example of a predator of other protists is *Lacrymaria* sp. This genus paralyses its prey with toxicysts prior to consuming it (Rosati et al., 2008). The genus *Platyophyra* is known to prey on bacteria and diatoms but additionally harbors symbiotic algae (Foissner and Kreutz, 1996), highlighting that classification of protists in trophic groups is not straightforward. This genus occurred in high abundance in jungle rubber. Further, *Vermamoeba* sp. and *Acanthamoeba* sp. frequently occurred in jungle rubber; both function as bacterial grazers with *Acanthamoeba* sp. a surface feeder (Saleem et al., 2016) potentially also feeding on algae (Marciano-Cabral and Cabral, 2003).

**Photoautotrophic protists**, traditionally termed algae and occurring in the sunlit uppermost soil layers, increased in richness and abundance in plantation systems benefitting from the more open canopy. In addition to increased canopy openness photoautotrophic protists may benefit from weed control in plantations contributing to increased sunlight reaching the soil surface. Further, the application of fertilizers is likely to favor the growth of photoautotrophs and to alter their community structure (Gilbert et al., 1998). Notably, Chlorophyta, i.e., green algae, are well adapted to harsh environmental conditions and disturbances as indicated by their frequent occurrence in deserts (Lewis et al., 2005). Thereby, Chlorophyta are well adapted to thrive in plantations with Chlorophyceae and Trebouxiophyceae dominating the photoautotrophs in plantations. As representatives of these groups, *Chrysochromulina* sp. and *Prymnesium* sp. were among the top ten most abundant OTUs in rainforest and jungle rubber, respectively. Species of these genera in marine systems are known to produce toxins with hemolytic, ichthyotoxic, and cytotoxic properties, affecting other algae and protists (Nielsen et al., 1990; Schmidt and Hansen, 2001; Fistarol et al., 2003). By producing toxins *Prymnesium parvum* may even kill its own predator, *Oxyrrhis marina*, and by consuming it switching to a phagotrophic lifestyle (Tillmann, 2003). This again highlights that the positioning of protists into trophic groups is not straightforward and this also applies to photoautotrophs.

**Symbionts** typically are tightly linked to host species, although the linkage may vary in space and time (Martin and Schwab, 2012). Although less than 1% of the total OTUs in our study were classified as symbionts only one of the OTUs could be ascribed to genus level, i.e., *Saccinobaculus* sp., an endosymbiont living in the hindgut of cockroaches (Heiss and Keeling, 2006). This genus only occurred in rainforest and jungle rubber, where cockroaches reach a high species richness (Mumme et al., 2015). The other OTUs were identified as Syndiniales, endosymbionts of ciliates, algae and other protists (Hoek et al., 1995). Indeed, the species richness of Syndinales increased with the species richness of phagotrophs and photoautotrophs, which dominate in plantations, likely reflecting increased host availability in plantations. However, certain Syndiniales in marine systems are known to be parasitic (Guillou et al., 2008), calling for careful interpretation of these findings.

**Parasites** are closely linked to their host species resembling symbionts, however, they detrimentally affect them (Martin and Schwab, 2012). Parasitic protists reach high abundance and species richness, and may strongly influence animals, fungi and plants as well as other protists, although this is mainly documented for marine systems (Skovgaard, 2014; de Vargas et al., 2015) including deep-sea hydrothermal vents (Moreira and López-García, 2003). However, recent studies suggest that this also applies to soils (Dupont et al., 2016; Geisen, 2016; Mahé et al., 2017). Notably, both groups of parasites identified, i.e., animal and plant parasites, reached higher abundance and species richness in rainforest as compared to plantation systems, matching the higher abundance and species richness of soil invertebrates and plants in rainforest as indicated by RDA. However, in addition to lower host availability, adverse environmental conditions in plantations may contribute to lower abundance and species richness of parasites in plantations, e.g., increased light intensity in plantations may detrimentally affect parasites, as exposure to UV may kill cysts of Eimeriidae (Thomas et al., 1995) causing coccidiosis in animals. Despite that, OTUs belonging to the family Eimeriidae were the most abundant OTUs of animal parasites in rubber and oil palm plantations. By contrast, the most abundant animal parasite protists in the more natural land-use systems included *Gregarina* sp., parasites of cockroaches (Clopton and Gold, 1996) and earwigs (Clopton et al., 2008).

The ever present *Eurychasma* sp., an oomycote with broad host range (Muller et al., 1999), was the most abundant plant parasite in each of the land-use systems. Peronosporomycetes, causative agents of downy mildew (Palti and Cohen, 1980), were less abundant in the more intensively managed land-use systems. This might be linked to the increased light intensity as red light inhibits the sporulation of *Peronospora* spp. (Cohen et al., 2013). *Polymyxa* sp., known to infect wheat and other crop species (Thompson et al., 2011; Ketta et al., 2012; Xu et al., 2018), formed part of the more abundant plant parasites in oil palm plantations. However, although *Polymyxa* sp. may infect a wide range of host species (Legrève et al., 2000), its effect on oil palms is unknown.

## Conclusion

Applying amplicon sequencing of the 18S rRNA gene of eDNA this study for the first time provided insight into the relative abundance and diversity of protists in rainforest and tropical agro-ecosystems. The results suggest that overall protist species richness is reduced in rubber plantations. By contrast, however, the community structure of protists is strongly affected by the conversion of rainforest into plantation systems with the relative abundance and relative species richness of the individual trophic groups responding differently. The abundance and in part also the species richness of phagotrophs, photoautotrophs and symbionts increased due to conversion of rainforest into plantation systems, whereas both abundance and species richness of parasites declined. Symbionts generally contributed little to protist abundance and species richness. Notably, within trophic groups individual taxa generally responded in a similar way, suggesting that trophic groups of protists reflect general patterns in changes in the structure of the micro-decomposer food web with conversion of rainforest into plantation systems.

## Author Contributions

GS, NB, and NE performed the laboratory work. GS, VK, and DS analyzed the data. GS drafted the manuscript. VK and SS designed the study and revised the first draft. All authors contributed to revising later drafts of the manuscript.

## Conflict of Interest Statement

The authors declare that the research was conducted in the absence of any commercial or financial relationships that could be construed as a potential conflict of interest.
