# Supplementary figures and images for: Changes in Trophic Groups of Protists With Conversion of Rainforest Into Rubber and Oil Palm Plantations
Source: Front Microbiol. 2019 Feb 12;10:240. doi: 10.3389/fmicb.2019.00240 (PMC6380168; doi:10.3389/fmicb.2019.00240)

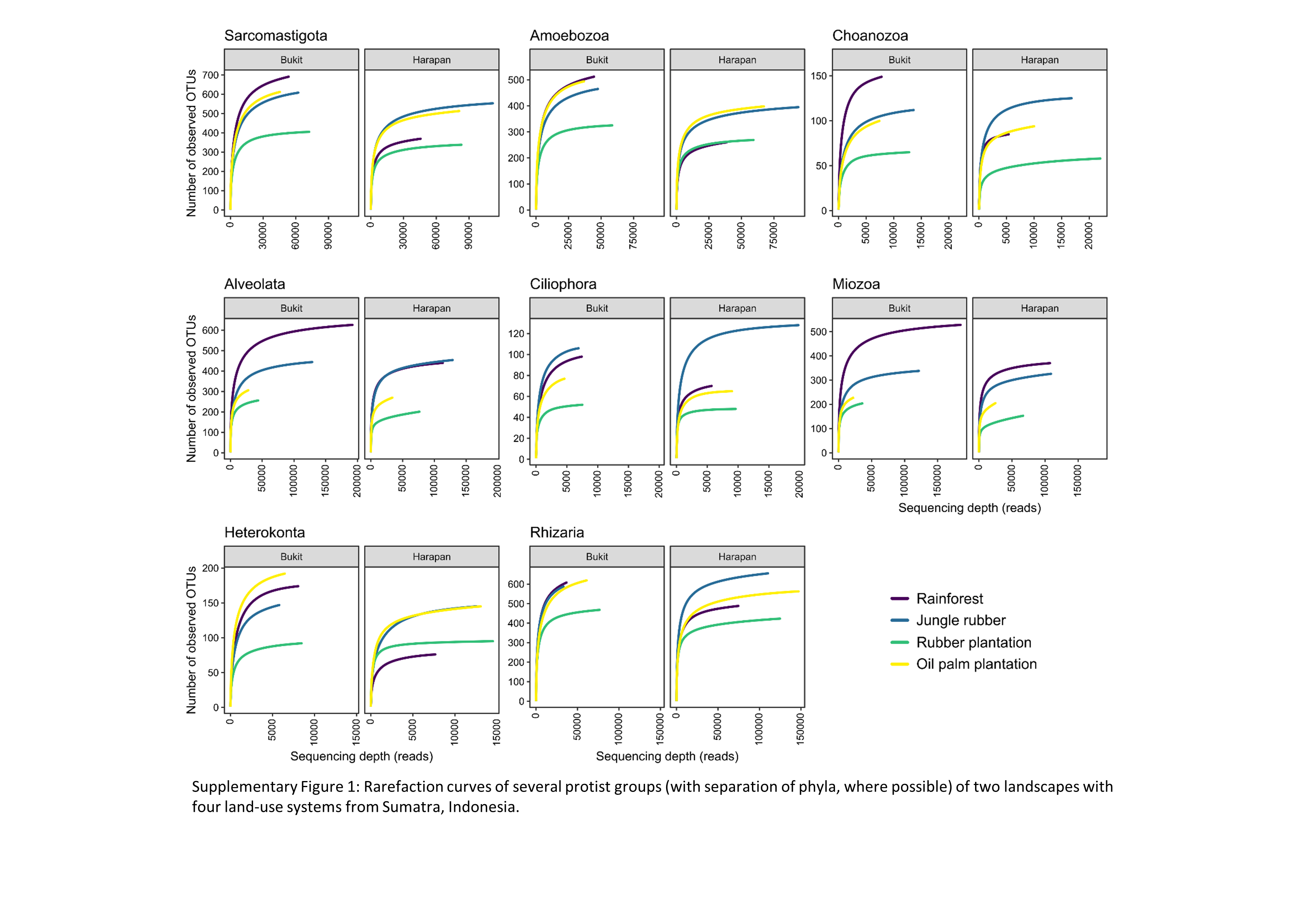

Supplement: Supplementary file 1 [file Image_1.TIFF]
